# Homœopathy—All about It

**Published:** 1895-03

**Authors:** 


HOMŒOPATHY: All about it; or the principle of cure. By John H. Clarke, M. D., editor of *The Homœopathic World*, etc., London. The Homœopathic Publishing Co., 12 Warwick Lane, Paternoster Row, E. C. 1894. Cloth, price, one shilling.

This charming little book of about one hundred pages should be in the hands of every physician. He should have extra copies to present to inquiring patients and skeptical adversaries.

In his exposition of the subject the author says on page 29: "Homœopathy theorizes about nothing. In its materia medica it admits no theories, no explanations of action, but only the *facts*."

Here is a statement worthy to be considered an aphorism; to be continually quoted to every caviler; to be continually impressed upon every hesitating follower, who assumes only the name of the new school and practices only the methods of the old school.

A fine defense of Hahnemann's doctrine of Psora is given on pages 40, 41, and 42. He says: "The Psora doctrine of Hahnemann is practically the same as the doctrine of certain French authorities who ascribe a great variety of chronic diseases to what they call a herpetic diathesis, that is to say, a morbid state of the organism liable to manifest itself on the skin by an itching vesicular eruption."

The inconsistency of the old school is exhibited here when they carp at "psora" and admit "herpetic diathesis." This indicates "what's in a name."

In his explanation and defense of infinitesimals the author quotes from Helmholtz the opinion that when the earth was in a nebulous state, "it would require several millions of cubic miles of such matter to weigh a single grain."

He quotes Darwin's experiments with the fly-catching plant Drosera or sun dew. Darwin found that certain salts of ammonia in solution caused the leaves to turn inward. Diluting his solutions more and more he at last found that it would respond to a solution containing only one twenty-millionth of a grain of the crystallized salt!

In speaking of the spread of Homœopathy the learned author says: "Considering that Homœopathy is only eighty-four years old and its founder has been dead little over sixty years, the extension of the system throughout the world does not leave very much to complain of."

Speaking of what it has done, he says: "Homœopathy has abolished bleeding from general practice and has done away with wholesale mercurializings." * * *

"At the present moment there are some fifteen thousand duly qualified medical practitioners openly practicing acccording to Hahnemann's method. How many more there are practicing Homœopathy secretly, for fear of the medical scribes and pharisees it is impossible to say; but their numbers cannot be small."

He tells the allopathic school with refreshing plainness, that the cause of quackery is their inefficiency, and adds that homœopathists are only a little troubled in this way.

The book winds up with a series of aphorisms on the law and the dose.

